# CD1-restricted T cells: are unconventional allies the key to future TB vaccines?

**DOI:** 10.3389/fimmu.2025.1629466

**Published:** 2025-07-10

**Authors:** Matthew Milton, Salah Mansour

**Affiliations:** ^1^ National Institute for Health and Care Research (NIHR) Biomedical Research Centre, School of Clinical and Experimental Sciences, Faculty of Medicine, University of Southampton, Southampton, United Kingdom; ^2^ Institute for Life Sciences, University of Southampton, Southampton, United Kingdom

**Keywords:** CD1, T cells, infection, tuberculosis, unconventional T cells, lipids, TB vaccine

## Abstract

CD1-restricted T cells constitute an unconventional arm of immunity that recognises lipid antigens, a feature particularly pertinent to *Mycobacterium tuberculosis* (Mtb), a pathogen with a lipid-rich cell wall. Unlike classical MHC-restricted responses, CD1-mediated lipid antigen presentation includes donor-unrestricted T cell responses, offering a promising pathway for universally protective tuberculosis (TB) vaccines. This review explores the biology of CD1 isoforms, the functional diversity of CD1-restricted T cell subsets, and their roles in TB immunity. We discuss Mtb’s lipid antigens, mechanisms of CD1 trafficking and antigen presentation, immune evasion strategies, and emerging translational insights. By highlighting key knowledge gaps and future directions, we argue that harnessing CD1-restricted T cells could unlock novel vaccine strategies against the world’s leading infectious killer.

## Introduction

In 2023, 10.8 million people fell ill with tuberculosis (TB), and 1.25 million died, making it the world’s leading cause of death from an infectious disease. The causative agent, *Mycobacterium tuberculosis* (Mtb), is estimated to infect around a quarter of the global population (1). Although TB primarily affects the lungs, it can also manifest in other parts of the body (2). The disease is highly contagious, disproportionately affects those living in poverty, and is costly and time-consuming to treat, all of which contribute to its immense global health burden (3). Compounding these challenges, the emergence of antibiotic-resistant Mtb strains poses a growing threat to TB control efforts (2).

Host immune responses to TB have traditionally focussed on peptide antigens presented by MHC class I and class II molecules, which are well studied in the context of conventional T cell immunity. However, these molecules are encoded by some of the most polymorphic genes in the human genome (4), resulting in substantial inter-individual variability in immune responses to Mtb (5, 6).

Mtb has a complex and lipid-rich cell wall, known as the mycolyl-arabinogalactan-peptidoglycan complex (7). Around 6% of the Mtb genome is dedicated to lipid metabolism (8), and lipids constitute approximately 40% of the cell envelope by weight (9). This unusual lipid composition underpins the pathogen’s virulence and resistance to antibiotics (10–15). Indeed, some of the most effective TB drugs, such as isoniazid, act by inhibiting Mtb lipid biosynthesis (8).

Importantly, these lipid components of the Mtb cell wall can be presented by CD1 molecules, a family of non-classical antigen-presenting molecules, to activate CD1-restricted T cells and initiate antimicrobial immune responses (8). Unlike MHC molecules, CD1 proteins are virtually non-polymorphic, meaning that the responses they elicit are genetically unrestricted and shared across the population (16, 17). This feature makes CD1 an attractive target for broadly effective TB vaccines, capable of overcoming the genetic variability that hampers conventional MHC-restricted vaccine approaches (18).

Despite their potential, CD1-restricted T cells remain underexplored in TB research, in part due to the technical challenges associated with studying them. However, harnessing these unconventional T cell responses may offer a novel strategy to enhance the efficacy of future TB vaccines (19). CD1-restricted T cell activation in TB appears to occur via two distinct but potentially complementary mechanisms. The first involves direct recognition of mycobacterial lipid antigens, such as mycolic acid, glucose monomycolate (GMM), or phosphomycoketides, presented by CD1 molecules on infected antigen-presenting cells (20–22). The second involves infection- or inflammation-induced remodelling of host lipid metabolism, leading to enhanced presentation of stimulatory self-lipids by CD1 and activation of autoreactive T cells (23). For instance, Toll-like receptor (TLR) signalling has been shown to promote the presentation of endogenous lipids such as sulfatide and GM1 (23), and in the case of CD1d-restricted iNKT cells, Brennan et al. (2011) demonstrated that microbial sensing by antigen-presenting cells (APCs) can induce self-lipid switching that drives T cell activation even in the absence of microbial antigens (24). Clarifying the relative importance of these pathways is essential for rational vaccine design and for maximising the potential of CD1-targeted immunity.

## Mechanisms of lipid antigen presentation by CD1 molecules

CD1 molecules are a family of non-polymorphic, non-classical MHC class I-like antigen-presenting molecules encoded on chromosome 1. They are specialised for the presentation of both self- and foreign lipid antigens to T cells. Based on sequence similarities, CD1 isoforms are classified into three groups: group 1 molecules (CD1a, CD1b, and CD1c), which are expressed exclusively by antigen-presenting cells (25) and cortical thymocytes, where they likely contribute to thymic selection of CD1-restricted T cells (26). CD1d, the sole member of group 2, is expressed across a broader range of immune cells (27). In contrast, CD1e, the only group 3 molecule, is restricted to intracellular compartments. CD1e localises to late endosomal compartments and facilitates lipid loading onto other CD1 isoforms (28). Structurally, CD1 molecules share homology with MHC class I molecules. Each CD1 molecule comprises a heavy chain with α1 and α2 domains forming the antigen-binding groove, structured as two antiparallel α-helices over a β-pleated sheet, and an immunoglobulin-like α3 domain with a transmembrane region and a short cytoplasmic tail anchoring the molecule to the membrane. Like MHC class I molecules, CD1 molecules associate non-covalently with β2-microglobulin. Lipid antigens bind within deep hydrophobic channels, with their polar headgroups exposed at the solvent interface for recognition by the T cell receptor (TCR). Differences in the size and shape of the antigen-binding grooves among CD1 isoforms facilitate the presentation of a wide range of lipid antigens (29–35).

CD1 molecules are synthesised in the endoplasmic reticulum (ER), where they associate with β2-microglobulin and undergo glycosylation, promoting interaction with ER chaperones such as calnexin, ERp57, and calreticulin (36–38). During biosynthesis, CD1 molecules bind endogenous lipids; some of these lipids stabilise the molecule, while others may be antigenic (39, 40). Following trafficking through the Golgi apparatus, CD1 molecules are transported to the plasma membrane.

Once at the cell surface, most CD1 molecules (excluding CD1a) are internalised via a clathrin-dependent pathway mediated by adaptor protein complex 2 (AP2), which recognises a tyrosine-based motif within their cytoplasmic tails (41–44). In contrast, CD1a lacks a tyrosine-based motif and is internalised independently of clathrin and AP2, through a Rab22a- and ADP-ribosylation factor 6 (ARF6)-dependent mechanism (45). After internalisation, CD1a and CD1c recycle predominantly through the early endocytic system back to the plasma membrane. CD1b and CD1d also recycle but, owing to differences in their sorting motifs, can additionally engage adaptor protein complex 3 (AP3) within sorting endosomes, facilitating trafficking through late endosomal and lysosomal compartments (46–48). CD1c can access both early and late endocytic pathways, giving it the most widespread distribution among CD1 isoforms within the endosomal system (41).

Endocytic compartments are enriched with exogenous lipids delivered via macropinocytosis (49), mannose receptors (50), langerin (51), and the low-density lipoprotein receptor (52). Within these compartments, internalised CD1 molecules encounter a variety of endogenous and exogenous lipid antigens. Lipid exchange is facilitated by CD1e and saposins, small non-enzymatic proteins found in lysosomes (28, 53–55). Thus, internalisation and trafficking through the endosomal system are crucial for the acquisition and presentation of lipid antigens (30). However, emerging evidence suggests that CD1a, possibly due to the more open structure of its binding groove, may also facilitate lipid exchange directly at the cell surface under neutral pH conditions (56, 57).

T cell responses to Mtb infection are unusual in that multiple T cell subsets recognise lipid antigens, many of which are derived from the Mtb cell wall and are presented by CD1 molecules (58). It is thought that the distinctive composition of the Mtb cell wall, together with the intracellular lifestyle of the bacilli, converges with CD1 loading pathways to promote lipid antigen presentation (26, 58, 59). Accordingly, CD1-restricted lipid presentation is considered a key element in the initiation and modulation of immune responses against Mtb.

## CD1-restricted T cells in tuberculosis immunity

Both αβ and γδ T cells have been shown to recognise lipid antigens presented by CD1 molecules. Despite the non-polymorphic nature of the CD1 system, the repertoire of CD1-restricted TCRs in humans is highly diverse (60–63). Increasing evidence highlights the importance of both αβ and γδ T cell subsets in host immune responses to Mtb infection (64–67).

Although αβ T cells are far more frequent in the blood, γδ T cells have gained significant research interest, particularly in the context of infection (68). γδ T cells normally account for approximately 4% of circulating T cells (69), but during infections such as TB, they can expand dramatically, representing up to 50% of the peripheral T cell pool (70–74). In fact, γδ T cells constitute the highest frequency of Mtb-reactive T cells in human peripheral blood (75). Hoft et al. (1998) demonstrated that γδ T cells were the most dramatically expanded population following stimulation of PBMCs from Bacille Calmette-Guérin (BCG)-vaccinated individuals with mycobacterial antigens. These γδ T cells also exhibited helper functions, supporting mycobacteria-specific CD4+ and CD8+ T cell responses (76). Moreover, γδ T cells have been shown to promote dendritic cell maturation, further linking them to the orchestration of both adaptive and innate immunity (77).

Human Vδ1 T cells have been reported to recognise all CD1 isoforms (64, 65, 78–85) whereas Vδ2 T cells predominantly recognise butyrophilins due to their TCRs usually containing the canonical Vγ9 chain (86–89). While Vδ2 T cells dominate the γδ T cell compartment in the blood of healthy individuals (90), TCR sequencing studies reveal that during active TB, the proportion of Vδ1 T cells increases markedly, resulting in codominance of Vδ1 and Vδ2 populations (16). In the lungs of TB patients, the γδ T cell repertoire is often highly skewed, dominated by locally expanded Vδ1 T cell clones (16). Given their abundance, elucidating the functional roles of Vδ1 T cells could significantly enhance our understanding of protective immunity to TB. Furthermore, due to their potent cytotoxicity and ability to exhibit immunological memory, Vδ1 T cells represent an attractive target for next-generation TB vaccine strategies (16, 65, 91–95).

Pioneering studies by Porcelli et al. (1998) provided direct evidence of CD1-restricted T cell responses. From healthy donor samples, they generated two T cell lines, BK6 (expressing an αβTCR) and IDP2 (expressing a γδTCR), both of which lacked CD4 and CD8 expression. Both lines could lyse the MOLT-4 T cell line in a CD1-dependent but MHC-independent manner (64). Lysis by BK6 was blocked by anti-CD1a antibodies, while lysis by IDP2 was blocked by anti-CD1c antibodies, demonstrating restriction by CD1a and CD1c, respectively. Blocking experiments confirmed that responses were TCR-mediated through the TCR-CD3 complex. Moreover, both T cell lines lysed mouse hybridoma and rhabdomyosarcoma cell lines transduced to express CD1a or CD1c, respectively, further confirming CD1 restriction (64).

Subsequent work by Rosat et al. (1999) described the generation of two CD8+ αβTCR-expressing T cell lines, CD8–1 and CD8-2, by stimulating PBMCs with Mtb lysates. CD8–1 specifically lysed CD1c-transfected target cells pulsed with Mtb lysates, while CD8–2 specifically lysed CD1a-transfected targets, with responses inhibited by blocking antibodies against CD1c and CD1a, respectively (96). Lysis was dependent on Mtb-derived lipid antigens, as no lysis occurred when target cells were pulsed with non-mycobacterial lysates or left untreated. Additionally, CD8–1 and CD8–2 secreted IFN-γ and TNF-α in response to Mtb antigen, but not Th2 or regulatory cytokines such as IL-4 or IL-10 (96). However, it is important to note that these responses were measured against Mtb lysate-pulsed APCs rather than live Mtb-infected cells, which may present distinct antigens.

Sieling et al. (2000) generated three CD4+ αβTCR-expressing T cell lines (LCD4.1, LCD4.2, and LCD4.3) from the skin lesions of leprosy patients (97). These T cells released IFN-γ in response to *Mycobacterium leprae* sonicate-pulsed dendritic cells (DCs), but not untreated DCs. Blocking experiments demonstrated that LCD4.1 responses were CD1c-restricted, while LCD4.2 and LCD4.3 were CD1b-restricted. Antigen specificity studies revealed that LCD4.2 recognised phosphatidylinositol mannoside and LCD4.3 recognised mycolic acid. Importantly, anti-CD4 blocking antibodies inhibited responses of MHC class II-restricted control T cells, but not LCD4.1 or LCD4.3, indicating that CD4 co-receptor engagement is not essential for CD1-restricted T cell activation (97).

By the early 2000s, strong evidence supported a role for CD1-restricted T cells in the immune response to Mtb. However, it remained unclear whether these cells expanded following Mtb exposure or differed in frequency between healthy and TB-infected individuals. Using PBMCs from PPD-positive and PPD-negative individuals, Ulrichs et al. (2003) demonstrated that T cells from PPD-positive individuals exhibited greater proliferation and IFN-γ secretion in response to Mtb lipid extracts, and these responses were largely CD1-dependent (98). CD3+ cell depletion abrogated IFN-γ production, confirming T cell involvement. Notably, CD1-restricted T cell responses were reduced or absent in active TB patients, suggesting that effective CD1-mediated immunity may be important for controlling Mtb infection, or that CD1-restricted T cells might migrate into infected lung tissue during active disease. Immunomagnetic separation further revealed that these responses were stronger in CD4+ compared to CD8+ T cells (98).

Kawashima et al. (2003) extended these findings by demonstrating that following BCG vaccination, CD8+ but not CD4+ T cells mounted CD1-restricted IFN-γ responses against BCG-infected dendritic cells. Responses by CD4+ T cells were dependent on MHC class II and unaffected by anti-CD1 blockade (99). Given that CD4+ T cell responses are essential for effective TB immunity (100–107), optimising TB vaccines to elicit robust CD4+ CD1-restricted memory T cell responses may be critical for achieving durable protection.

One of the major obstacles to studying CD1-restricted T cells is that mice lack group 1 CD1 molecules. To address this, Felio et al. (2009) developed a transgenic mouse model expressing human CD1a, CD1b, and CD1c. In response to Mtb infection, these mice generated CD1-restricted T cell responses characterised by cytotoxicity, IFN-γ production, and memory formation (108).

Although most mammals possess group 1 CD1 genes, muroid rodents (mice and rats) are an exception (109–112). Thus, guinea pigs, which express CD1b and CD1c, have also been used as an alternative model. Hiromatsu et al. (2002) showed that guinea pigs immunised with Mtb lipids generated CD1-restricted T cell responses that were cytotoxic and exhibited immunological memory (113). In subsequent studies it was shown that immunised guinea pigs had reduced lung pathology as well as reduced bacterial burden in the lungs and spleen following Mtb infection (114). Together, these findings highlight the importance of CD1-restricted T cells in immunity to Mtb and underscore their potential as targets for future TB vaccine development. Additional details on CD1 genes across various mammalian species are provided in **Table 1**.

**Table 1:** Number of CD1 genes across different mammalian species.

Common name	Binomial species name	Genome	Number of genes					
			CD1a	CD1b	CD1c	CD1d	CD1e	Total CD1
Alpaca	*Vicugna pacos*	vicPac2	1	1	1	1	1	5
Bonobo	*Pan paniscus*	panPan1	1	1	1	1	1	5
Chimpanzee	*Pan troglodytes*	panTro4	1	1	1	1	1	5
Dog	*Canis lupus*	CanFam3	9	1	1	1	1	13
Elephant	*Loxodonta africana*	loxAfr3	1	2	1	1	1	6
Horse	*Equus caballus*	equCab2	9	2	2	1	2	16
Human	*Homo sapiens*	hg38	1	1	1	1	1	5
Megabat	*Pteropus vampyrus*	pteVam1	3	1	1	0	1	6
Microbat	*Myotis lucifugus*	myoLuc2	17	2	0	5	2	26
Mouse	*Mus musculus*	mm10	0	0	0	2	0	2
Panda	*Ailuropoda melanoleuca*	ailMel1	8	1	1	1	1	12
Pig	*Sus scrofa*	susScr3	2	1	1	1	2	7
Rabbit	*Oryctolagus cuniculus*	oryCun2	5	2	0	1	2	10
Rhesus macaque	*Macaca mulatta*	rheMac3	2	1	1	1	1	6

CD1 gene counts were identified using BLAST-based genome searches. Data are adapted from Reinink et al. (2016) which systematically analysed CD1 gene families across mammalian genomes (115).

## CD1a-restricted responses to infection

CD1a is highly expressed by Langerhans cells (LCs) and plays an important role in generating T cell responses in the skin and at other mucosal sites (51, 116). Unlike other CD1 isoforms, CD1a may be capable of lipid exchange at the plasma membrane under neutral pH conditions, possibly due to the more open structure of its binding groove. CD1a molecules are also stabilised by exogenous lipids present in serum (56, 57).

LCs are a specialised subset of dendritic cells critical for initiating and regulating immune responses in the skin. Hunger et al. (2004) compared the expression of dendritic cell markers on LCs and conventional DCs. LCs exhibited higher expression of langerin (CD207), CD58, and CD1a, whereas DCs expressed higher levels of CD86, CD11c, CD1b, and HLA-DR; expression of CD14, CD80, CD83, and CD1c was similar between the two populations (51). Using CD1a+ LC-like DCs derived from leprosy patients, two CD1a-restricted αβTCR-expressing T cell clones, B2.1 and B2.11, were generated. Both clones were double-negative (DN) for CD4 and CD8, and they proliferated in response to CD1a+ LC-like DCs pulsed with *Mycobacterium leprae* extracts. Their responses were specifically inhibited by anti-CD1a, but not anti-CD1b or anti-CD1c, blocking antibodies, confirming CD1a restriction (51).

Interestingly, B2.1 and B2.11 also responded to extracts from *M. tuberculosis*, *Mycobacterium smegmatis*, and *Mycobacterium phlei*, but not to extracts from *Mycobacterium avium*, *Nocardia*, *Aspergillus*, or *Rhodococcus* species, suggesting recognition of a specific exogenous lipid antigen present in a subset of bacterial species. These clones expanded and secreted IFN-γ when co-cultured with LC-like DCs but showed only limited responses to monocyte-derived DCs (MoDCs), highlighting the superior ability of LCs to stimulate CD1a-restricted T cell responses (51). Langerin is involved in pathogen sensing (117) and in the formation of Birbeck granules (118). Given the differences in langerin expression between LCs and DCs, Hunger et al. investigated its role in CD1a-restricted responses. Pre-treatment of LC-like DCs with anti-langerin antibodies before, but not after, pulsing with *M. leprae* extracts inhibited T cell proliferation, suggesting that langerin is involved in the uptake, processing, or presentation of lipid antigens (51).

In parallel, Moody et al. (2004) used a CD1a-restricted αβTCR transfected J.RT3-T3.5 T cell reporter line to screen Mtb lipid fractions for antigens (119). High-performance liquid chromatography and mass spectrometry identified a series of related stimulatory lipids. Further structural analysis using nuclear magnetic resonance and mass spectrometry revealed the antigen as a lipopeptide, named didehydroxymycobactin, likely an intermediate in the mycobactin biosynthetic pathway of the Mtb cell wall. Importantly, Mtb-infected MoDCs, but not uninfected cells, could present this antigen to activate the CD1a-restricted reporter line, confirming that didehydroxymycobactin is naturally processed and presented during infection (119).

## CD1b-restricted responses to infection

Among the group 1 CD1 molecules, CD1b is unique in its ability to present lipid antigens with very long acyl chains, such as mycolic acid (120). CD1b-restricted T cells are the best characterised of all group 1 CD1-restricted populations, and extensive studies have established their role in responses to Mtb infection.

The first evidence of CD1 antigen presentation came from Porcelli et al. (1992), who generated a CD1b-restricted T cell line from αβTCR-expressing double-negative (DN) T cells cultured with Mtb extract-pulsed MoDCs. These T cells lysed Mtb-infected, CD1b-transfected C1R cells, but not cells transfected with CD1a, CD1c, or empty vectors, demonstrating CD1b restriction (121).

Building on this, Beckman et al. (1994) identified mycolic acid as a lipid antigen presented by CD1b using organic phase separation and T cell proliferation assays. A CD1b-restricted T cell clone, DN1, specifically recognised mycolic acid derivatives, including 6,6’-trehalosedimycolate, but not irrelevant lipids, suggesting TCR-mediated recognition (21). Further studies expanded the catalogue of CD1b-presented antigens. Sieling et al. (1995) identified lipoarabinomannan as a mycobacterial lipid recognised in a CD1b-dependent manner by DN αβ T cells derived from leprosy patients and healthy donors, with these T cells capable of lysing antigen-pulsed monocytes and secreting IFN-γ (122).

Similarly, Stenger et al. (1997) showed that CD1b-restricted T cells from TB patients and healthy donors could lyse Mtb-infected macrophages in a CD1-dependent manner. Distinct cytotoxic mechanisms were observed: DN T cells relied on Fas-FasL interactions, while CD8+ T cells used granule-mediated killing. Importantly, CD8+ T cells, but not DN T cells, significantly reduced intracellular Mtb growth, likely via granulysin secretion (123, 124).

The identification of specific mycobacterial lipid antigens continued with Moody et al. (1997), who demonstrated that the LDN5 T cell clone recognised GMM presented by CD1b (22). Gilleron et al. (2004) later characterised Ac2SGL, a sulfoglycolipid, as a potent CD1b-restricted antigen stimulating IFN-γ and granulysin secretion by CD8+ T cells, leading to reduced Mtb growth. Responses to Ac2SGL required endosomal processing and were absent in PBMCs from PPD-negative individuals, suggesting selective expansion with prior Mtb exposure (125). Layre et al. (2009) identified glycerol monomycolate (GroMM) as another CD1b-presented antigen using the Z5B71 T cell clone. IFN-γ responses to GroMM were observed in BCG-vaccinated and latent TB individuals, but absent in active TB, suggesting defective memory responses during disease (126).

Montamat-Sicotte et al. (2011) further demonstrated that mycolic acid-specific CD1b-restricted T cells were enriched in TB patients, including at the site of infection (bronchoalveolar lavage fluid), and persisted long after treatment, indicating durable memory responses. Interestingly, BCG vaccination alone did not generate strong mycolic acid-specific memory T cells, possibly due to differences in mycolic acid structure between Mtb and BCG strains (127–129).

The development of CD1b tetramers revolutionised the study of CD1b-restricted T cells. Kasmar et al. (2011) showed that GMM-loaded CD1b tetramers specifically stained the LDN5 T cell clone and rare T cells in TB patient PBMCs, which were predominantly CD4+ (130). Rhijn et al. (2013) used tetramers to isolate and characterise CD1b-GMM specific T cell clones. High-affinity clones, called germline-encoded mycolyl lipid-reactive (GEM) T cells, all shared a TRAV1-2–TRAJ9 α-chain signature and expressed predominantly CD4. TCR sequencing confirmed that both α- and β-chains contributed to antigen specificity. GEM T cells expanded in TB patients, supporting their role in immune responses to Mtb (131). In contrast, LDN5-like T cells, expressing TRAV17 and TRBV4-1, represented a second group of GMM-specific T cells with more diverse TCR usage and coreceptor expression (132).

Functional evidence for CD1b-mediated protection came from Busch et al. (2016), who showed that lipoarabinomannan-specific CD1b-restricted T cells from latent TB individuals inhibited Mtb growth in MoDCs, and that these T cells produced granulysin, a molecule essential for direct killing of Mtb (124, 133).

Most recently, Sakai et al. (2024) identified trehalose monomycolate (TMM) as a novel CD1b-presented antigen. Using CD1b tetramers and single-cell RNA and TCR sequencing, they showed that TMM-specific T cells upregulate cytotoxic molecules such as granzyme B, perforin, and granulysin. These T cells expanded in TB patients and recognised TMM from multiple mycobacterial species but required the trehalose headgroup for TCR recognition (134). Cryo-electron microscopy revealed the ternary structure of the CD1b-TMM-TCR complex, providing detailed molecular insights into lipid antigen recognition.

Finally, Zhao et al. (2015) generated a transgenic mouse model expressing human CD1a, CD1b, CD1c, and a DN1 TCR specific for mycolic acid. In this model, DN1 T cells reduced Mtb burden after adoptive transfer, highlighting the protective capacity of CD1b-restricted T cells during TB infection (135). Together, these findings establish CD1b-restricted T cells as key contributors to host defence against Mtb and highlight their potential as targets for next-generation TB vaccines.

## CD1c-restricted responses to infection

Among the group 1 CD1 molecules, CD1c is the most widely expressed and exhibits the broadest distribution throughout the endocytic system (25, 27, 41, 60, 66, 136, 137). This extensive trafficking enables CD1c to survey a diverse range of lipid antigens. Moreover, unlike other CD1 isoforms, CD1c lipid loading is independent of compartment acidification, a process that Mtb actively inhibits to evade phagocytic destruction, giving CD1c a potential advantage in infection settings (41, 138).

Both αβ and γδ T cells can recognise lipid antigens presented by CD1c. Despite the non-polymorphic nature of the CD1 system, the repertoire of CD1c-restricted TCRs is highly diverse (60–67).

Moody et al. (2000) first demonstrated that lymphocytes from individuals with prior Mtb exposure and positive PPD skin tests showed significantly greater proliferation and activation in response to synthetic isoprenoid glycolipids, structurally similar to Mtb antigens, in a CD1c-dependent manner. These findings provided the first evidence of CD1c-mediated lipid-specific memory T cell responses in infectious disease (66).

Building on this, Matsunaga et al. (2004) investigated the CD1c-restricted T cell line CD8-1 [previously described by Rosat et al. (96)]. They demonstrated that CD8–1 cells proliferated in response to CD1c-transfected C1R cells pulsed with Mtb or BCG whole lipid extracts, as well as with mannosyl-β-1-phosphoisoprenoids, a family of Mtb lipid antigens presented by CD1c (66). Disruption of the pks12 gene in Mtb abrogated the synthesis of mannosyl-β-1-phosphoisoprenoids, and lipid extracts from pks12 knockout strains failed to activate CD8–1 T cells. Mass spectrometry confirmed the absence of mannosyl-β-1-phosphoisoprenoids in the mutant strains, establishing pks12 as essential for their biosynthesis (8). Among these antigens, mannosyl-β1-phosphomycoketide (MPM) is now recognised as a major target for CD1c-restricted T cell responses in Mtb-exposed individuals (20).

Further work by Ly et al. (2013) expanded the repertoire of known CD1c-presented Mtb lipids. Using fractionated lipid extracts and the DN6 CD1c-restricted T cell line as a reporter, they identified a novel antigen, C32 phosphomycoketide (PM), a fully saturated C32 alkylphosphate structurally related to MPM (20). DN6 T cells were strongly activated by PM-pulsed MoDCs, but not by extracts from pks12-deficient Mtb, confirming PM as a natural mycoketide antigen. Notably, DN6 responded to both PM and deglycosylated forms of MPM, suggesting that antigen processing by APCs, involving removal of β-linked mannose units, can influence CD1c-restricted T cell recognition. Plate-bound CD1c experiments further confirmed distinct modes of recognition by different T cell clones (20).

Currently, PM and MPM remain the only two natural CD1c-presented Mtb lipid antigens that have been clearly identified (20, 60, 66, 139, 140). Further antigen discovery will likely be important for optimising TB vaccines aimed at targeting CD1c-restricted T cell responses.

To address antigen stability, Reijneveld et al. (2021) synthesised an MPM analogue, MPM-3, designed to resist enzymatic hydrolysis during antigen processing. *In vitro* immunisation with MPM-3 expanded MPM-specific T cells that demonstrated dual reactivity towards both MPM and MPM-3. These findings suggest that MPM-3 could serve as a more stable vaccine component for inducing robust CD1c-restricted T cell responses (141).

## CD1d-mediated responses to mycobacteria

CD1d presents lipid antigens to natural killer T (NKT) cells, including invariant NKT (iNKT) cells and some Vδ1 T cells (79, 142). NKT cells are a distinct population of αβTCR-expressing T cells that co-express natural killer (NK) markers such as CD94 and CD161 (143). In humans, iNKT cells, also referred to as type 1 NKT cells, are defined by expression of a semi-invariant Vα24-Jα18 TCR (144, 145), whereas type 2 NKT cells possess a more diverse TCR repertoire (146). The synthetic glycosphingolipid α-galactosylceramide (α-GalCer), originally isolated from a marine sponge, binds CD1d and strongly activates iNKT cells by engaging their TCR with high affinity (147–149). The development of CD1d-α-GalCer tetramers enabled detailed characterisation of iNKT cells in both mice and humans (150, 151). Importantly, α-GalCer does not activate type 2 NKT cells, providing a selective tool for studying iNKT biology. Much of our understanding of CD1d-restricted immunity stems from iNKT cell research, largely because both CD1d and iNKT cells are conserved across mice and humans (152), unlike group 1 CD1 molecules, which are absent in murine models.

While iNKT cells were first investigated in the context of cancer, where α-GalCer treatment reduced tumour metastases and improved survival in mouse models (153), they have also been implicated in protection against Mtb infection. Chackerian et al. (2002) showed that α-GalCer administration prolonged survival and reduced lung bacterial burden in Mtb-infected CD1d-sufficient, but not CD1d-deficient mice, demonstrating a CD1d-dependent protective effect (154).

Subsequent studies identified microbial lipid antigens presented by CD1d. Fisher et al. (2004) demonstrated that phosphatidylinositol mannoside (PIM), a lipid isolated from BCG, could stimulate murine iNKT cells via CD1d presentation. Using CD1d-transfected B cell lymphoma cells pulsed with PIM, they observed IFN-γ secretion from Vα14-Jα281 transgenic mouse splenic T cells, a response abrogated by anti-CD1d blocking antibodies. These responses were absent with untransfected B cells, confirming CD1d restriction. Moreover, CD1d-PIM tetramers could stain murine iNKT cells similarly to CD1d-α-GalCer tetramers (155).

Investigations in humans revealed that iNKT cell clones stained by both CD1d-PIM and CD1d-α-GalCer tetramers also secreted IFN-γ and lysed CD1d-transfected HeLa cells pulsed with PIM. No lysis was observed when untransfected HeLa cells were used, confirming CD1d-restricted recognition. These findings identify PIM as a mycobacterial lipid antigen capable of activating human iNKT cells (155). Beyond recognition of foreign lipids, iNKT cells can also be activated by stress-induced self-lipid antigens. Brennan et al. (2011) showed that TLR stimulation of dendritic cells triggers lipid remodelling, promoting presentation of endogenous agonists on CD1d and enhancing iNKT activation in the absence of microbial lipid antigens. This ‘self-lipid switching’ provides a key mechanism by which innate immune cues can modulate CD1d-restricted T cell responses during infection (24). Functional studies further demonstrated a role for iNKT cells in controlling Mtb infection. Sada-Ovalle et al. (2008) showed that murine iNKT cells upregulated the activation marker CD69 upon contact with Mtb-infected macrophages, but not uninfected controls. Splenocytes from wild-type, but not iNKT-deficient, mice were able to reduce Mtb growth in infected macrophages. Furthermore, splenocytes failed to control Mtb growth when infected macrophages lacked CD1d, demonstrating the necessity of CD1d-mediated presentation. Pure iNKT cell lines were sufficient to inhibit Mtb growth when cultured with infected macrophages, and adoptive transfer of iNKT cells into irradiated, Mtb-infected mice significantly reduced bacterial burden in both lungs and spleen (156).

Collectively, these findings suggest that CD1d-restricted iNKT cells can contribute to anti-Mtb immunity. However, conflicting results exist. One study found no significant difference in survival between wild-type and CD1d-deficient mice infected with Mtb, suggesting that CD1d-restricted responses may not be essential for protection (157). Thus, while evidence supports a role for iNKT cells in immunity to Mtb, their contribution may vary depending on the infection model and experimental conditions.

## Autoreactivity is an intrinsic feature of CD1 biology

An unusual feature of CD1-restricted T cells is their frequent autoreactivity. Although thymic selection minimises self-reactivity in conventional T cells, CD1-autoreactive T cells are nevertheless abundant in healthy individuals (26).

De Jong et al. (2010) showed that T cells from all 14 healthy donors tested exhibited reactivity towards CD1a-expressing K562 cells, whereas responses to CD1b, CD1c, or CD1d were less common. Blocking with anti-CD1a antibodies confirmed CD1a restriction. CD1a-autoreactive T cells, comprising approximately 2% of circulating T cells, were predominantly CD4+, produced IFN-γ, IL-22, and sometimes IL-13, but often lacked IL-2 production. Many expressed cutaneous lymphocyte antigen (CLA), suggesting skin homing. T cells isolated from skin biopsies similarly showed CD1a reactivity, with stronger responses when stimulated by Langerhans cells (158).

De Lalla et al. (2011) independently confirmed that CD1 autoreactivity is relatively frequent. Single-cell cloning revealed that around 10% of both CD4+ and DN αβT cells were self-reactive to CD1 molecules, predominantly CD1a and CD1c. TCR repertoire analysis showed high diversity among self-reactive clones, contrasting with the invariant TCRs of iNKT cells (159). CD1c-autoreactive T cells were functionally heterogeneous: CD4+ clones were more likely to secrete TNF-α, DN clones secreted GM-CSF, and some clones produced both Th1 and Th2 cytokines. Importantly, CD1a- and CD1c-autoreactive clones demonstrated cytotoxicity against target cells expressing their cognate CD1 isoforms without exogenous antigen, indicating intrinsic autoreactive killing potential (159). Beyond classical Th1 cytokines, CD1-autoreactive T cells can secrete a broad range of effector molecules, including GM-CSF, IL-13, IL-22, and IL-5 (158–160). For example, CD1c- and CD1b-autoreactive T cell clones have been shown to produce polyfunctional responses that include both Th1 and Th2 cytokines, and in some cases, GM-CSF and IL-22, which can enhance antigen presentation, promote monocyte recruitment, and contribute to mucosal barrier integrity (159, 160). IL-13 and IL-5, though traditionally associated with Th2 responses, may modulate inflammation or tissue repair in TB lesions. These findings suggest that CD1-restricted T cells may play diverse immunomodulatory roles during TB infection, beyond direct cytotoxicity or classical macrophage activation.

Despite their prevalence, CD1-restricted self-reactive T cells rarely cause pathology, suggesting regulatory mechanisms are in place. Nevertheless, associations with autoimmune diseases have been reported. CD1c+ antigen-presenting cells infiltrate lesions in Graves’ disease and Hashimoto’s thyroiditis, and T cells capable of lysing CD1c+ targets have been isolated from thyroid tissue (161). In systemic lupus erythematosus (SLE), DN T cells reactive to CD1c can produce IL-4 and IFN-γ, and may support IgG production by CD1c+ B cells (162). In rheumatoid arthritis, synovial fluid contains increased numbers of activated CD1c+ dendritic cells that stimulate CD4+ T cells, although CD1c restriction was not definitively proven (163). Autoreactivity has also been implicated in multiple sclerosis (MS). Shamshiev et al. (1999) found increased frequencies of T cells reactive to brain-derived glycolipids in MS patients. Two T cell clones recognised monosialo-ganglioside GM1 presented by CD1b, and responses were blocked by anti-CD1b antibodies, implicating CD1b in autoreactive responses against myelin components (164).

Recent mechanistic studies have further clarified how CD1 autoreactivity may be regulated. De Jong et al. (2014) demonstrated that CD1a-autoreactive T cells, such as clone BC2, recognised CD1a loaded with endogenous lipids from the epidermis, including squalene from sebaceous glands. These findings suggest that spatial separation of self-lipids, for example, lipids located beyond T cell access in healthy skin, helps prevent inappropriate activation (165). Moreover, Betts et al. (2017) showed that the contact dermatitis agent 2,4-dinitrochlorobenzene (DNCB) activates CD1a-autoreactive T cells, suggesting environmental exposures can trigger pathological autoreactive responses. In functional studies, DNCB-treated CD1a+ APCs stimulated a polyfunctional cytokine response from autoreactive T cells (166).

Guo et al. (2018) engineered K562 cells to express high levels of CD1c and used them to stimulate peripheral blood T cells without exogenous antigen (167). Activated T cells upregulated CD154 and showed enrichment of TRBV4+ TCRs, specifically TRBV4-1. Responses were blocked by anti-CD1c antibodies. When TRBV4-1+ TCRs were transduced onto Jurkat cells, they responded to CD1c-expressing targets, confirming CD1c autoreactivity (167).

Further mechanistic insights have come from studies of lipid antigen structure. Cotton et al. (2021) found that sphingomyelins with long unsaturated acyl chains (e.g., 42:2 sphingomyelin) inhibit CD1a-TCR interactions by protruding from the antigen-binding groove and sterically blocking TCR engagement, whereas shorter chain sphingomyelins are permissive. Thus, specific endogenous lipids can negatively regulate CD1a autoreactivity (23).

Structural studies also support a model of direct CD1 recognition without lipid co-recognition. Wun et al. (2018) solved the structure of an autoreactive CD1c-restricted TCR bound to CD1c presenting a fully sequestered endogenous lipid. The TCR contacted CD1c itself rather than the presented lipid, consistent with “CD1-as-antigen” recognition. These findings explain how high frequencies of autoreactive CD1c-restricted T cells can exist without constant activation, provided regulatory mechanisms are intact (136).

Several broader mechanisms are also thought to limit autoreactive responses:

- Inhibitory lipid loading: Endogenous lipids with bulky head groups, such as phosphatidylcholine or sphingomyelin, may block TCR access to CD1 molecules (23).
- Tissue-specific CD1 expression: Although CD1c is expressed on B-cells, group 1 CD1 molecules are expressed relatively sparsely in the peripheral blood, restricting opportunities for autoreactive encounters (136, 168).
- TCR internalisation: In CD1b-transgenic mice, autoreactive CD1b-restricted T cells showed reduced surface TCR expression compared to wild-type mice, suggesting that downregulation of TCR levels may suppress autoreactivity *in vivo* (169).

Together, these mechanisms contribute to immune tolerance, preventing frequent autoreactivity from manifesting as autoimmune disease. Importantly, autoreactivity does not necessarily equate to autoimmunity, and controlled self-reactivity may even have physiological roles yet to be fully defined.

## CD1-autoreactive T cells: are they really so evil?

Despite their association with autoimmune diseases, CD1-autoreactive T cells have been conserved throughout human evolution, suggesting they may play beneficial roles in immunity.

CD1c is expressed on several haematological malignancies, including B cell acute lymphoblastic leukaemia (B-ALL) and acute myeloid leukaemia (AML) in both adults and children (170). Lepore et al. (2014) isolated CD1c-autoreactive T cell clones from healthy donors and found that these clones secreted GM-CSF and IFN-γ in response to CD1c-transfected THP1 and C1R cells in a CD1c-dependent manner. Lipid extraction and fractionation of THP1 cells identified a stimulatory lipid, later determined by mass spectrometry as methyl-lysophosphatidic acid (mLPA). Synthetic mLPA analogues loaded onto recombinant CD1c similarly activated CD1c-autoreactive T cells, confirming mLPA as an endogenous immunogenic ligand.

Functionally, mLPA-specific T cell clones secreted IFN-γ in response to co-culture with CD1c+ AML cells, but not with healthy monocytes. Despite lower CD1c expression on some AML cells compared to monocytes, stronger T cell activation was observed against the leukaemic cells, suggesting increased mLPA presentation. Similarly, B-ALL cells induced greater IFN-γ secretion compared to normal B cells despite similar CD1c expression levels. Direct quantification showed mLPA accumulation was significantly higher in leukaemic cells than in healthy cells.

mLPA-specific T cells preferentially killed B-ALL and AML cells while sparing most normal B cells and monocytes. Killing was CD1c-dependent, as blocking antibodies abrogated cytotoxicity. *In vivo*, mLPA-specific T cells prolonged survival in an immunodeficient mouse model grafted with CD1c+ MOLT-4 leukaemia cells. Furthermore, healthy donor T cells transduced with mLPA-specific TCRs acquired the ability to recognise and respond to CD1c+ target cells, demonstrating the therapeutic potential of CD1c-autoreactive TCRs (170). Beyond cancer, CD1-autoreactive T cells have also been shown to contribute to antimicrobial immunity. Vincent et al. (2005) generated 15 group 1 CD1-restricted T cell clones by stimulating CD4-depleted T cells with CD1-expressing MoDCs and lipid extracts from Mtb, *E. coli*, or *Yersinia enterocolitica*. All clones were CD8+ αβTCR+ T cells that proliferated in response to CD1-expressing MoDCs without additional stimulation, demonstrating autoreactivity. These T cells were highly cytotoxic against CD1-transfected HeLa and C1R cells, as well as MoDCs. Cytokine profiling showed expression of IFN-γ, GM-CSF, IL-5, and IL-13, and functional responses were abrogated by CD1-blocking antibodies. Moreover, CD1a- and CD1b-restricted TCRs transduced into Jurkat cells conferred both self-reactivity and enhanced responses to microbial lipids, highlighting dual specificity for self- and foreign antigens (160).

Similarly, Roy et al. (2016) identified CD1c-restricted γδ T cells through CD1c-PM tetramer staining of human PBMCs. Sorted lines predominantly expressed the Vδ1 TCR chain, confirming that Vδ1 cells are the main γδ T cell population recognising CD1c (65, 78). Upon transduction of Vδ1+ TCRs into Jurkat cells, spontaneous activation was observed, indicating low-level autoreactivity towards endogenous CD1c ligands. These TCR-transduced Jurkat cells responded more strongly when stimulated with PM, demonstrating dual reactivity to both endogenous and microbial lipid antigens. Binding studies confirmed that different lipids modulated TCR engagement: some TCRs bound more strongly to CD1c presenting self-lipids, while others preferred microbial lipids (78).

Following on from previous work (169), Bagchi et al. (2016) demonstrated that a CD1b-autoreactive T cell line, which recognises phospholipids, secreted IL-2 in response to plate-bound CD1b protein loaded with lipids extracted from normal cells, indicating autoreactivity. However, IL-2 secretion was significantly higher when CD1b was loaded with lipids extracted from the T lymphoblast cell line MOLT-4, suggesting that cancer cell-derived lipids are more immunogenic. Furthermore, these T cells were able to lyse CD1b-transfected, but not wild-type, murine RMA-S T cell lymphoma cells, confirming CD1b-restricted recognition and cytotoxicity (171).

**Figure 1:**
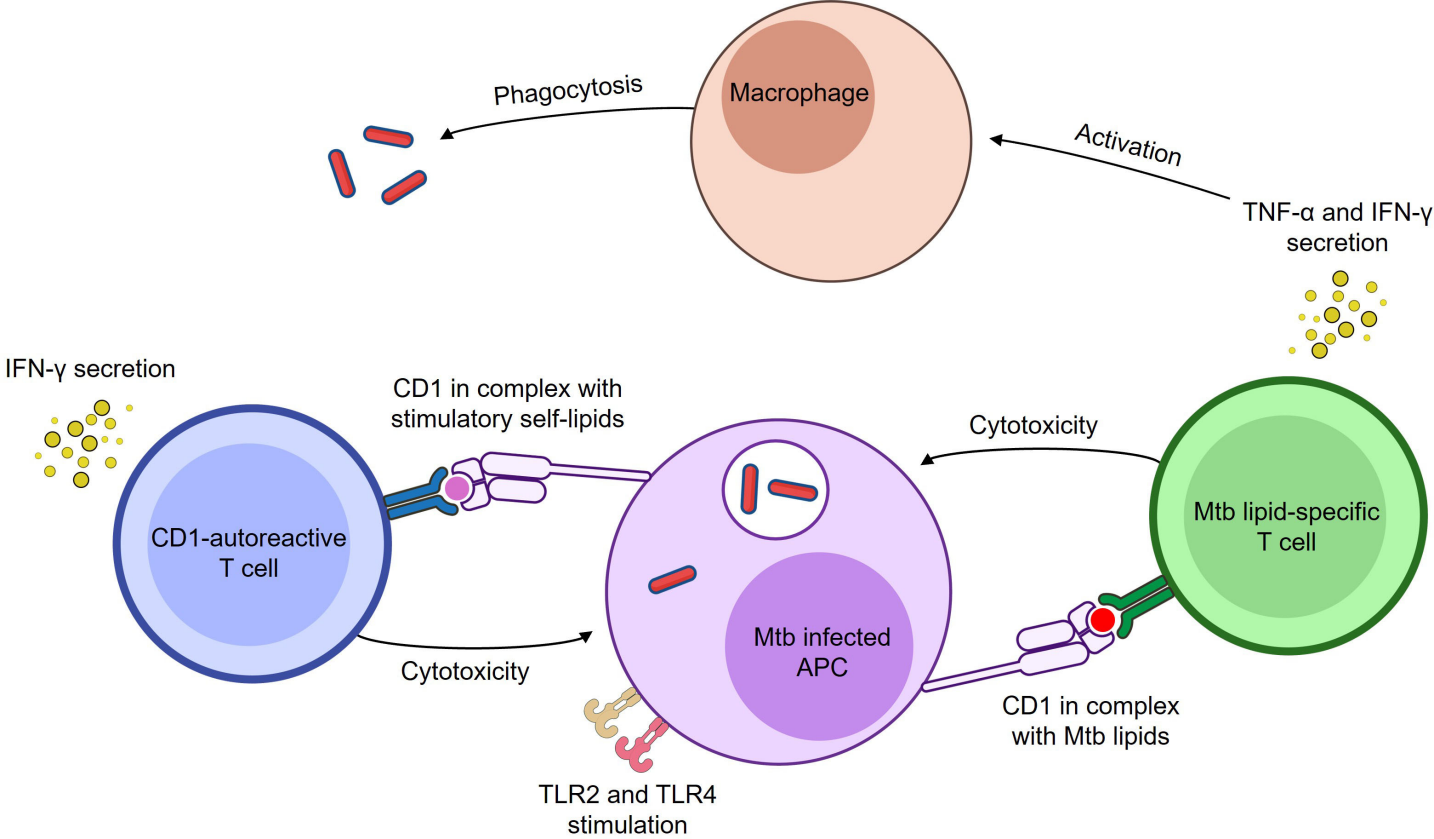
Both Mtb lipid-specific and CD1-autoreactive T cells respond to Mtb infection. Both Mtb lipid-specific and CD1-autoreactive T cells respond to Mtb infection. Mtb lipid-specific T cells become cytotoxic and secrete IFN-γ and TNF-α (96, 113, 123, 134) in response to Mtb lipid antigens presented by CD1 molecules on infected APCs. CD1-autoreactive T cells can also become cytotoxic and secrete IFN-γ in response to either Mtb lipid or stress-induced self-lipid antigens presented by CD1 molecules on infected APCs (78, 160). While Mtb can gain access to the cytosol, it predominantly resides in the phagosome, where it is sensed by innate receptors and processed for antigen presentation. TLR signalling enhances these responses by upregulating CD1 expression, costimulatory molecules, and presentation of stimulatory self-lipids. Specifically, TLR2 and TLR4, which recognise Mtb lipoproteins and cell wall components, are shown in the figure. These receptors bridge innate sensing of Mtb with adaptive CD1-restricted T cell responses. Secreted IFN-γ and TNF-α can stimulate macrophages to enhance antimicrobial functions (172, 173). Image created using BioArt.

In follow-up experiments, mice were inoculated with either wild-type or CD1b-transfected RMA-S tumour cells, alongside CD1b-autoreactive T cells. On day 14, mice were sacrificed, and tumour size was measured. Tumour growth was significantly reduced in mice that received CD1b-transfected RMA-S cells and CD1b-autoreactive T cells, suggesting that these T cells can mediate anti-tumour immunity in a CD1b-dependent manner. In contrast, no reduction in tumour size was observed in mice inoculated with wild-type RMA-S cells, confirming that the protective effect was specifically mediated by CD1b recognition (171). Collectively, these findings suggest that CD1-autoreactive T cells, rather than being solely pathogenic, may have beneficial roles in immune surveillance. Based on this evidence, CD1-autoreactive T cells could contribute to host defence against both tumours and infections and may play a previously underappreciated role in the immune response to infectious diseases such as tuberculosis (**Figure 1**).

## TLR signalling modulates CD1-autoreactive T cell responses during infection

TLR stimulation can influence the functional responses of CD1-autoreactive T cells, linking innate immune sensing to adaptive lipid-specific immunity. De Libero et al. (2005) investigated two CD1a-restricted T cell clones specific for sulfatide and two CD1b-restricted clones specific for monosialo-ganglioside GM1 (172). When co-cultured with immature DCs infected with *E. coli*, *B. subtilis*, *S. aureus*, or BCG, all four clones secreted IFN-γ. Similarly, stimulation of DCs or CD1-transfected THP1 cells with the TLR4 agonist LPS or the TLR2 agonist Pam3Cys significantly enhanced IFN-γ production by these CD1-autoreactive clones. Responses were CD1-dependent, as blocking antibodies abrogated T cell activation, and were not observed in MHC class II-restricted or γδ T cell clones under identical conditions (172).

Mechanistically, LPS and Pam3Cys stimulation modestly increased CD1 and co-stimulatory molecule expression (B7.1, CD40) on DCs and THP1 cells. More strikingly, infection or TLR stimulation induced increased synthesis of the self-lipid antigens sulfatide and monosialo-ganglioside GM1, suggesting that infection-driven changes in lipid metabolism enhance CD1-restricted T cell activation by elevating the abundance of stimulatory self-lipids (172). This mechanism is mirrored in the CD1d–iNKT cell axis, where TLR activation of dendritic cells drives lipidome remodelling and presentation of stimulatory self-lipids, enabling iNKT activation in the absence of microbial antigens (24). Earlier work from the same group showed that microbial infection can activate iNKT cells via CD1d-mediated presentation of endogenous lipids, further supporting TLR-induced self-lipid switching as a general mechanism of CD1-restricted immunity (174). Zeissig et al. (2012) similarly demonstrated that hepatitis B virus infection alters hepatocyte lipid composition to generate CD1d-presented lysophospholipids, triggering NKT cell activation (175). Together, these findings highlight a broader mechanism by which pathogen sensing promotes autoreactive T cell responses through enhanced self-lipid presentation.

Li et al. (2011) further explored this phenomenon using a transgenic mouse model expressing group 1 CD1 molecules and a CD1b-autoreactive TCR (169). Treatment of bone marrow-derived DCs with Pam3Cys, LPS, or *Listeria monocytogenes* infection resulted in heightened secretion of IFN-γ and IL-17A from CD1b-autoreactive T cells compared to untreated controls. Following *in vivo* challenge with *Listeria monocytogenes*, CD1b-autoreactive T cells upregulated the activation marker CD69 and contributed to a reduced bacterial burden in the liver and spleen, compared to non-transferred controls. These findings suggest that TLR2 and TLR4 signalling can amplify CD1b-autoreactive T cell responses during infection, promoting pathogen clearance (169). This highlights a model in which inflammation-driven upregulation of CD1 expression and stress lipid synthesis can activate CD1-autoreactive T cells, even in the absence of strong pathogen-derived lipid presentation. For a more extensive discussion on how TLR pathways intersect with CD1-restricted T cell immunity, we refer readers to the comprehensive review by Moody et al. (2006) (176).

## Mtb infection influences CD1 expression

During the late 1990s and early 2000s, a series of studies investigated how mycobacterial infection affects CD1 molecule expression by APCs.

Stenger et al. (1998) infected human adherent mononuclear cells (AMNCs) treated with GM-CSF and IL-4 with live Mtb and measured group 1 CD1 expression by flow cytometry. No significant changes were observed at 4 hours post-infection; however, by 24 hours, reduced staining of all group 1 CD1 isoforms was evident, and by 48 hours, expression was undetectable (177). Quantitative RT-PCR confirmed a substantial decrease in group 1 CD1 mRNA levels in infected cells compared to controls. Notably, infection with heat-killed Mtb did not reduce CD1 expression, indicating that live bacilli are necessary for this effect. Using a transwell system, the authors demonstrated that soluble factors alone were insufficient to mediate CD1 downregulation, suggesting that direct interactions between live Mtb and host cells are required (177).

Giuliani et al. (2001) further explored the effects of mycobacteria on CD1 expression. They found that infection with live BCG inhibited the GM-CSF-induced upregulation of group 1 CD1 molecules, particularly CD1b. In contrast to Stenger et al., heat-killed BCG also suppressed CD1b expression, attributed to alternative mRNA splicing mechanisms. Interestingly, using a transwell system, they observed that soluble factors secreted by BCG-infected AMNCs could reduce CD1b expression in adjacent uninfected cells, suggesting a mechanism of bystander suppression not observed with Mtb (178).

Wen et al. (2013) later demonstrated that CD1c mRNA levels are reduced in PBMCs from TB patients compared to healthy controls (179). An inverse correlation between CD1c expression and miR-381-3p levels was identified, suggesting post-transcriptional regulation. Binding of miR-381-3p to the 3’ untranslated region of CD1c was confirmed using a luciferase reporter assay. Overexpression of miR-381-3p in DCs decreased CD1c expression, while inhibition of miR-381-3p restored it. Furthermore, BCG infection increased miR-381-3p levels and decreased CD1c expression, effects reversible with miR-381-3p inhibition. Importantly, blocking miR-381-3p enhanced CD1c-restricted T cell responses to BCG, suggesting that miR-381-3p inhibitors could be therapeutically useful to improve vaccine-induced CD1-mediated immunity (179).

Given that mycobacteria have coevolved with innate immune systems (180), it is plausible that downregulating group 1 CD1 molecule expression represents an immune evasion strategy, limiting recognition by CD1-restricted T cells. These findings collectively suggest that modulation of CD1 expression by mycobacteria could impair host immunity, and that targeting these pathways could lead to improved vaccine strategies capable of eliciting more robust CD1-restricted memory responses (177–179). Given Mtb’s ability to modulate CD1 expression, it is essential that future vaccine strategies account for these evasion mechanisms.

In the following section, we review the current landscape of TB vaccine development and explore how targeting CD1-restricted immunity could offer new opportunities for protection.

## TB vaccine development

Developing a more effective vaccine remains one of the most promising strategies to control TB and limit the rise of antibiotic-resistant strains. The only currently available TB vaccine, BCG, contains an attenuated form of *Mycobacterium bovis* (181). Although BCG is widely administered and offers relatively high protection against childhood TB meningitis and miliary TB, it provides limited protection against pulmonary TB in adults and adolescents, the major drivers of Mtb transmission (18, 182–184). Furthermore, BCG is contraindicated in immunocompromised individuals, including those with untreated HIV infection, due to the risk of disseminated BCG infection (185).

Several new TB vaccine candidates have entered clinical trials in recent years. One strategy involves viral-vectored vaccines such as MVA85A, based on a modified vaccinia Ankara virus expressing Mtb antigen 85A. Despite encouraging preclinical data, MVA85A failed to provide protection in Phase IIb clinical trials in infants and adults, marking a major disappointment as the first new TB vaccine candidate to undergo an efficacy trial in over 80 years (186–188).

Subunit vaccines have also been explored. H56:IC31 is a fusion protein combining Ag85B, ESAT-6, and Rv2660c, the latter preferentially expressed during Mtb latency (189). In mice, a BCG prime followed by an H56:IC31 boost reduced lung bacterial burden after Mtb challenge (190). In cynomolgus macaques, H56:IC31 boosting after BCG vaccination delayed progression to active TB, extended survival, and reduced pathology (191). Early-phase human trials showed that H56:IC31 was well tolerated and induced robust IgG and antigen-specific CD4+ T cell responses in both Mtb-infected and uninfected individuals (192, 193). However, in a Phase IIb trial, although immunogenic, H56:IC31 unexpectedly showed higher TB incidence among vaccinees (5.8%) compared to placebo (3.4%) (194).

Another prominent candidate is M72/AS01E, a recombinant fusion protein vaccine combining the Mtb antigens PepA and PPE18. PepA is thought to function as a serine protease (195), while PPE18 may interact with TLR2, inducing immunosuppressive IL-10 responses (196–198), and promoting Mtb survival (199). However, PPE18 exhibits substantial structural variability across strains (200), raising concerns about antigenic consistency. The Phase IIb trial of M72/AS01E was hailed as a breakthrough by the WHO, demonstrating approximately 50% protection against progression to active TB three years post-vaccination (201). Nevertheless, injection-site reactogenicity led to delayed recruitment in some Phase II trials (202), and the moderate efficacy suggests further vaccine improvements are still needed.

One limitation of viral-vectored, subunit, and recombinant fusion vaccines is their narrow antigenic focus. None of MVA85A, H56:IC31, or M72/AS01E can generate CD1-restricted memory responses to Mtb lipids. However, future formulations could incorporate immunogenic lipid antigens to elicit CD1-restricted immunity. Morgun et al. (2023) developed a nanoparticle-based TB vaccine containing both mycolic acid and the protein antigen Ag85B. In mice, this formulation activated adoptively transferred DN1 T cells (specific for mycolic acid) and Ag85B-specific T cells *in vivo*. Additionally, human mycolic acid-specific T cells responded to the same nanoparticles *in vitro*, highlighting the potential of subunit vaccine platforms that combine lipid and protein antigens to elicit broad CD1- and MHC-restricted T cell responses (203).

Whole-cell vaccines offer broader antigen presentation, including lipid antigens (99). MTBVAC, a live attenuated Mtb strain with deletions in phoP and fadD26, genes essential for virulence lipid synthesis, is currently in Phase III trials (204–209). Another candidate, VPM1002, is a recombinant BCG expressing listeriolysin O from *Listeria monocytogenes* and lacking urease C. This enables phagosome acidification and cytosolic antigen release, enhancing immunogenicity (187, 210–216).

However, because both Mtb and BCG have been shown to downregulate CD1 expression and impair CD1-restricted T cell responses (177–179), MTBVAC and VPM1002 may still have limited capacity to induce optimal CD1-restricted memory. Identifying and reversing the mechanisms by which mycobacteria suppress CD1 expression could offer a route to improving future vaccines. It also remains unclear whether optimal CD1-restricted responses should be elicited by including defined mycobacterial lipids as vaccine immunogens, or by leveraging innate activation to drive self-lipid presentation and autoreactive T cell activation. Both approaches merit investigation.

Importantly, CD1 molecules are non-polymorphic, meaning immune responses to CD1-presented antigens are shared across genetically diverse human populations. Thus, targeting CD1-restricted responses may enable broader and more universal vaccine coverage (18). Furthermore, lipid antigens are less prone to mutational escape compared to peptides presented by classical MHC molecules, as alterations to essential lipid biosynthetic pathways often compromise bacterial viability (170).

Given the major advances in TB vaccine development in recent years, there is real hope that new immunisation tools capable of providing better global protection are within reach. Targeting CD1-restricted T cell responses offers a promising strategy to boost future vaccine efficacy against TB, the world’s leading infectious killer (1, 19).

## Conclusions

CD1-restricted T cells offer a compelling yet underutilised opportunity to transform TB vaccine development. Their capacity to recognise lipid antigens via non-polymorphic CD1 molecules allows for genetically unrestricted, population-wide immune responses (17, 18). While pathogen-specific (20–22, 66, 97, 119, 122, 125, 126, 129–134, 155) and autoreactive (78, 160, 169, 172) CD1-restricted T cells can both contribute to antimicrobial defence, Mtb’s ability to downregulate CD1 expression presents a key challenge (177–179). Current vaccines fail to engage lipid-specific memory responses, revealing a critical gap. Future strategies that incorporate immunogenic lipid antigens, restore CD1 expression (179), and selectively expand protective CD1-restricted T cell subsets may deliver the next leap in TB vaccine efficacy. Integrating CD1-targeted immunity could move us closer to durable, universal protection against TB.
